# Preparation of Fucoidan-Based Electrospun Nanofibers and Their Interaction With Endothelial Cells

**DOI:** 10.3389/fbioe.2021.739209

**Published:** 2021-09-06

**Authors:** Yiwen Chen, Huilin Zhu, Yuanping Hao, Zhanyi Sun, Peili Shen, Qihui Zhou

**Affiliations:** ^1^ Department of Stomatology, Institute for Translational Medicine, The Affiliated Hospital of Qingdao University, Qingdao University, Qingdao, China; ^2^ School of Stomatology, Qingdao University, Qingdao, China; ^3^ State Key Laboratory of Bioactive Seaweed Substances, Qingdao Bright Moon Seaweed Group Co., Ltd., Qingdao, China

**Keywords:** fucoidan, electrospun nanofibers, extracellular matrix, endothelial cells, biointerface, cell-material interface

## Abstract

Sulfated polysaccharide fucoidan (FD) is widely applied in biomedical applications owing to its outstanding bioactivities. In addition to the biochemical features, the architecture of biomaterials plays a critical role in tissue repair and regeneration. Particularly, nanofibers have elicited great interest due to their extracellular matrix-like structure, high specific surface area, and favorable biological properties. Herein, chitosan-modified FD/ultra-high molecular weight polyethylene oxide (UHMWPEO) nanofibers are developed *via* green electrospinning and electrostatic interaction for studying their interaction with endothelial cells. The appropriate solvent is screened to dissolve FD. The electrospinnability of FD/UHMWPEO aqueous solutions is greatly dependent on the weight ratios of FD/UHMWPEO. The incorporation of UHMWPEO significantly improves the electrospinnability of solution and thermo-stability of nanofibers. Also, it is found that there is good miscibility or no phase separation in FD/UHMWPEO solutions. *In vitro* biological experiments show that the chitosan-modified FD/UHMWPEO nanofibers greatly facilitate the adhesion of endothelial cells and inhibit the attachment of monocytes. Thus, the designed FD-based nanofibers are promising bio-scaffolds in building tissue-engineered blood vessels.

## Introduction

In the past few decades, marine polysaccharides have gained increasing attention in the area of diversified biomedical applications owing to their inherent (bio)physicochemical features, such as biocompatibility, biodegradability, favorable bioactive, biomechanical properties, and structural functionalities (Bidarra et al., 2014; Fernando et al., 2019; Hao et al., 2020; Jana et al., 2020; Yin et al., 2021; Zheng et al., 2021). Particularly, sulfated polysaccharide fucoidan, extracted from marine brown seaweed, has been well-known to possess various biological activities, e.g., antibacterial, antiviral, antioxidant, anticoagulant, anti-inflammatory, antitumor, antithrombotic, antifibrotic, and immunomodulatory activities, facilitating the generation of angiogenesis and fibrillar collagen matrix (Li et al., 2008; Senthilkumar et al., 2017; Oka et al., 2020; Yao et al., 2020). These unique characteristics make them remarkable candidates for blood vessel tissue engineering, which has not been examined closely.

Besides the biochemical properties, their biophysical structure can significantly mediate cell attachment, shape, viability, the differentiation or pluripotency of stem cells, and even tissue repair and regeneration (Li et al., 2018; Cui et al., 2020; Yu et al., 2020; Yu et al., 2021; Zhou et al., 2020; Liu et al., 2021; Yang et al., 2021a; Yang et al., 2021b). Recently, the development of nanofibrous materials has received increasing attention in tissue engineering and regenerative medicine due to their outstanding properties, such as their favorable biological properties, sufficient mechanical strength, highly porous mesh with interconnectivity, extremely high specific surface area, and aspect ratio (Zhou et al., 2015; Zhou et al., 2017; Kenry and Lim, 2017; Xue et al., 2019; Ahmadi et al., 2021). In addition, nanofibers can mimic the natural extracellular matrix (ECM) structure in the blood vessel and have been widely used as a blood vessel tissue-engineering scaffold (Xu et al., 2004; Devolder et al., 2011). In the recent 2 decades, the electrospinning technique has been widely used to prepare polymeric fibers with diameters typically ranging from tens of nanometers to several micrometers (Zhang et al., 2005; Xue et al., 2019; Daraeinejad and Shabani, 2021; Fetz et al., 2021; Peng et al., 2021). However, the electrospinning of fucoidan (FD) remains a challenge due to its low viscoelasticity and solubility issues. It was reported that other nature polymers [e.g., chitosan (CS), cellulose, sodium alginate, protein] with a small amount of ultra-high molecular weight polymer (UHMWP) [e.g., polyethylene oxide (PEO), polyvinyl alcohol (PVA), polyvinyl pyrrolidone (PVP)] can allow their preparation in nanofibers *via* electrospinning (Zhang et al., 2008; Li et al., 2015). In this sense, the combination of FD and UHMWP could also be considered to address the issue of spinnability.

**FIGURE 1:**
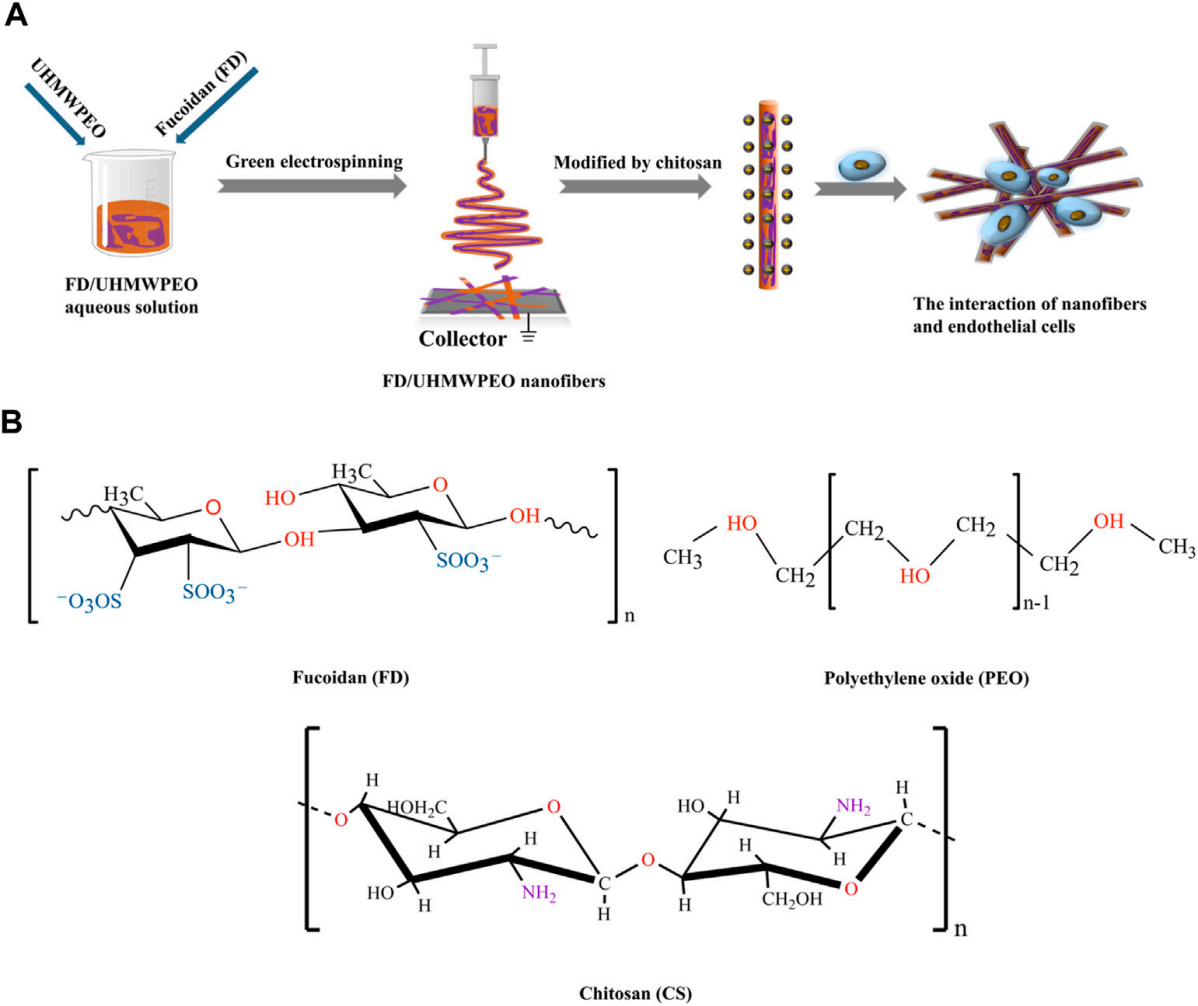
**(A)** Schematic diagram of the preparation of CS-modified FD/UHMWPEO nanofibers and their interaction with HUVECs. **(B)** Chemical structures of FD, CS, and PEO.

Vascular endothelial cells (VECs) are the predominant cell type and generate a continuous inner monolayer of blood vessels, which are responsible for regulating inflammation and vascular homeostasis in healthy blood vessels (Coultas et al., 2005). Also, the attachment of monocytes to VECs is vital for the occurrence of atherosclerosis and inflammation (Rajendran et al., 2013; Yang et al., 2019; Li et al., 2021a; Li et al., 2021b; Zong et al., 2021). Herein we hypothesize that FD-based nanofibers would be able to exhibit favorable physicochemical properties to mediate VEC responses in engineering vascular tissues. To test the hypothesis, FD/UHMWPEO nanofibrous films were fabricated using green electrospinning. Figure 1A displays the overall strategy to develop CS-modified FD/UHMWPEO nanofibers and their interaction with VECs. H_2_O and a small amount of UHMWPEO were selected as the solvent and co-spinning polymer for electrospinning of FD. Then, positively charged CS was selected to interact with negatively charged FD *via* the electrostatic interaction. The chemical structures of FD, UHMWPEO and CS used are shown in Figure 1B. The physicochemical features of FD-based nanofibers, i.e., morphology, crystallization, and thermal properties, were systematically tested by different characterization techniques. Further, FD-based nanofibers were seeded with human umbilical VECs (HUVECs) to investigate the effects of material physicochemical properties on cellular attachment and the adhesion of monocytes to HUVECs.

## Materials and Methods

### Materials

Fucoidan (FD, Mw = 276 kDa, sulfate: 29.65%) was provided by Qingdao Bright Moon Seaweed Group Co., Ltd. (Qingdao, China). UHMWPEO (Mv = ∼6,000,000 g/mol^−1^), chitosan (CS, Mv = 300 kDa and deacetylation degree ≥90%), and acetic acid (HAc, purity ≥99.8%) were supplied by Shanghai Macklin Biochemical Co., Ltd. (Shanghai, China). Both the human umbilical vein endothelial cells (HUVECs) and human acute monocytic leukemia cells (THP-1) were bought from the Shanghai Institutes for Biological Sciences (Shanghai, China). Dulbecco’s Modified Eagle Medium/Nutrient Mixture F-12, RPMI 1640 media, and fetal bovine serum were supplied by Biological Industries (Israel). FITC phalloidin and DAPI were provided by Solarbio (Beijing, China). Cell Counting Kit-8 was purchased from Absin Bioscience Inc. (China). Carboxyfluorescein diacetate succinimidyl ester was provided by MedChemExpress (Shanghai, China). Other chemical reagents were of analytical grade and used without further purification. Ultrapure water used in all experiments was obtained with a Milli-Q apparatus (Millipore, Bedford, MA, USA).

### Preparation of Electrospun FD-Based Nanofibers

The FD aqueous solutions were doped with a small amount of UHMWPEO (i.e., FD/UHMWPEO = 100/0, 98/2, 97/3, 96/4, 95/5, 94/6, 93/7, 92/8, 91/9, and 90/10). The mixed solutions were stirred for ∼6 h at room temperature prior to processing to ensure thorough mixing. The solution was loaded into a 20 ml plastic syringe attached with a 25-gauge blunt-ended needle as the spinneret which was charged at a high electric potential of 10–15 kV by a high voltage power supply (Tianjin Dongwen High Voltage Power Supply Plant, China). The solution feeding rate (0.3–1 ml/h) was precisely controlled by a syringe pump (Baoding Longer Precision Pump Co., Ltd., China). The FD-based nanofibers were collected onto an aluminum foil-covered collector placed 15 cm away from the needle tip. Electrospinning processes were performed on a horizontal electrospinning setup at 20–25°C with an ambient humidity of 30–35%.

### Modification of FD-Based Nanofibers by CS

FD-based nanofibers prepared from FD/PEO (90/10) were particularly selected for modification with CS. First, 1% CS was dissolved in an aqueous mixed solvent system consisting of 30, 60, and 90 wt% HAc, respectively. The FD-based nanofibers were immersed in the CS/HAc solution for ∼60 s. All modified samples were dried for 2–3 days in a vacuum oven (DZF-6050AB, Beijing, China) at 35°C to remove any potential residual solvent.

### Characterization

The morphological structure of the prepared nanofibers was observed using a scanning electron microscope (SEM) (VEGA3, TESCAN, Czech) operated at an acceleration voltage of 8–10 kV. Prior to observation, samples were sputter-coated with gold for 120 s to increase the electronic conductivity. The mean diameter of nanofibers was identified by randomly detecting at least 50 fibers from various SEM images for each type of sample using Image J software.

The rheometer (MCR301, Anton Paar, China) equipped with a parallel plate (20 mm) was used to measure the viscous property of FD/PEO aqueous solutions.

A Nicolet iN10 FTIR spectrometer (Thermo Fisher Scientific, Waltham, MA, USA) was used to characterize Fourier transform-infrared (FTIR) spectra of the samples over the range of 500–4,000 cm^−1^ at a scanning resolution of 2 cm^−1^ during 32 scans.

X-ray diffraction (XRD) spectroscopy was performed by DX2700 (Dandong, China) to measure the crystal structures of nanofiber samples. The samples were tested between 10 and 80° (2θ) at a scanning rate of 0.05° (2θ) per min operating with voltage 40 kV and current 30 mA equipped with Cu Kα radiation (*λ* = 1.5418 Å).

Thermogravimetric analysis on the nanofiber samples was conducted in a thermogravimetric analyzer (NETZSCH, Germany) at a scan range from 0 to 800°C with continuous nitrogen flow.

Differential scanning calorimetry (DSC, TA, USA) was used to measure the thermal properties of the electrospun FD-based nanofibers. A nitrogen atmosphere (flow rate = 50 ml/min) was used throughout. All samples were first quenched to -80°C with liquid nitrogen and then heated at a rate of 10°C/min to 180°C.

### Cellular Assays

HUVECs (passage: 3–5) were cultured in Dulbecco’s Modified Eagle Medium/Nutrient Mixture F-12 (Biological Industries, Israel) supplemented with 10% fetal bovine serum (Biological Industries, Israel) and 1% Penicillin-Streptomycin Liquid (Biological Industries, Israel) in a humidified incubator of 5% CO_2_ at 37°C. THP-1 cells were cultured in RPMI 1640 media (Biological Industries, Israel) supplemented with 10% FBS in a humidified 37°C and 5% CO_2_ incubator. THP-1 cells were used in the following experiments.

All substrates (Ø14 mm) were immersed into 75% ethanol for 2 min and then irradiated with UV for 1 h, placed in 24-wells, and washed by PBS. After that, HUVECs were incubated on the substrates in 24-well plates at a density of 3 × 10^4^ cells/well for cell adhesion. All plates were stored in an incubator at 37°C and 5% CO_2_ for 24 h. Then, HUVECs were fixated by 4% paraformaldehyde (Solarbio, Beijing, China) for 20 min. Subsequently, the cell membrane was permeabilized with 0.5% Triton X-100 (Sigma) solution for 3 min. Finally, the cells were stained by FITC phalloidin and DAPI for 30 and 10 min, respectively (Solarbio, Beijing, China). The images were captured by Fluorescence Microscopy (Nikon A1 MP, Japan).

HUVECs were seeded onto the sterilized substrate (Ø14 mm) in 24-well plates at a density of 5 × 10^4^ cells/well for forming cell monolayers. After 1 day, THP-1 cells (1.5 × 10^5^ cells/well) stained by Carboxyfluorescein diacetate succinimidyl ester (CFSE, MCE, China) were seeded onto HUVEC monolayer, and co-cultured for 4 h. Afterward, each well was washed with PBS three times and counted the number of THP-1 adhered by HUVECs using the Fluorescence Microscopy (Nikon A1 MP, Japan).

### Statistical Analysis

All data were expressed as mean ± SD. Statistical analysis was performed using Origin 9.0. All the data were analyzed using one-way analysis of variance (ANOVA) with Tukey’s test to determine differences between groups. A value of *p* < 0.05 was considered to be statistically significant.

## Results and Discussion

### Solubility of FD in Various Solvents

It was demonstrated that the selection of solvent is critical to determine material solubility, viscoelasticity, electrical conductivity and electrospinnability of the solution, as well as the productivity and morphology of nanofibers (Zhou et al., 2013; Casasola et al., 2014). However, no studies have been performed to find out which solvents FD could dissolve in. In our study, FD was first dispersed into 11 solvents as shown in Table 1 under magnetic stirring at room temperature. After 12 h, it was found that FD was only dissolved in the water (Table 1), which formed a hazel homogeneous solution (data not shown). The maximum solubility of FD in the water at room temperature is 10%. When water was heated to 40°C, FD dissolved faster and the amount of dissolved FD significantly increased. Therefore, in the following experiment water was used as a solvent to prepare FD nanofibers *via* electrospinning. Also, water-based electrospinning, also named “green electrospinning,” has several advantages of being environmentally friendly, non-toxic, and non-flammable. It was reported that organic solvents remaining in the fibers had a negative effect on cellular adhesion and proliferation both *in vitro* and *in vivo* (Mooney et al., 1996; Lv et al., 2018). The water-based electrospinning strategy here for preparing FD nanofibers is a safe and versatile route to numerous applications in biology, medicine, and pharmacy.

**TABLE 1:** The solubility of FD in different solvents.

H_2_O	DCM	EA	DMSO	TCM	DMF	Diox	CCl_4_	CAN	Hex	THF
**+**	−	−	−	−	−	−	−	−	−	−

DCM, Dichloromethane; EA, Ethyl acetate; DMSO, Dimethyl Sulphoxide; TCM, Trichloromethane; DMF, Dimethyl Formamidine; Diox, Dioxane; CCl_4_, Carbon Tetrachloride; CAN, Acetonitrile; Hex, Hexyl hydride; THF, Tetrahydrofuran. “−” means insolubilization; “+” means solubilization.

### Preparation of FD-Based Electrospun Nanofibers

**FIGURE 2:**
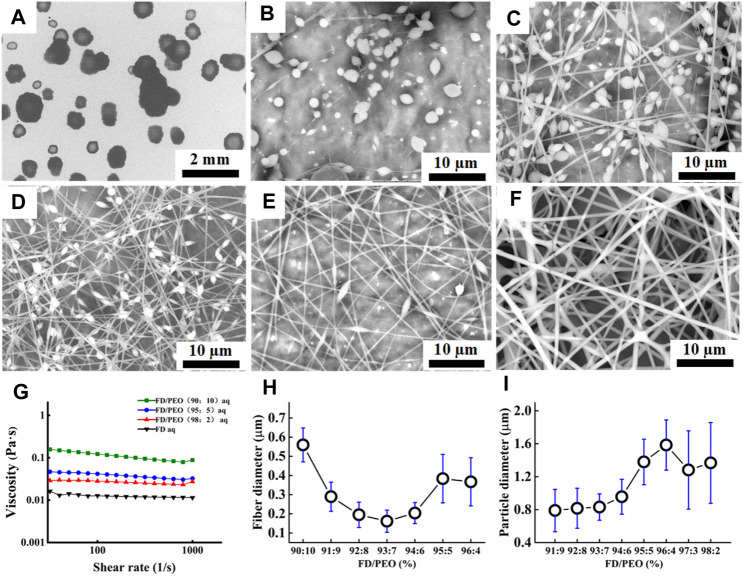
**(A–F)** SEM images of FD/PEO electrospun nanofibers with different weight ratios of FD/PEO (i.e., 100/0, 98/2, 97/3, 93/7, 91/9, and 90/10). **(G)** The viscosity of FD/PEO solutions with different weight ratios. **(H, I)** Dependence of fiber diameter and microbead size on different weight ratios of FD/PEO, respectively.

To obtain the adequate viscosity of FD solution, the maximum FD concentration (10% w/v) at room temperature was used in the following experiment. However, when 10% w/v FD aqueous solution was used for electrospinning, only droplets were formed as shown in Figure 2A, probably because the used FD solution still did not have enough viscosity.

As reported, the electrospinnability of naturally derived polymer solutions can be greatly improved by introducing a small amount of UHMWPEO (Zhang et al., 2008; Li et al., 2015). As shown in Figure 2B, with the decrement of the weight ratios of FD/PEO from 100:0 to 98:2, FD/PEO microbeads were fabricated. When the mass ratio of FD/PEO was further decreased from 97:3 to 91:9, the nanofibers with bead-string morphology were generated, the microspheres were elongated, and the average diameter of nanofibers decreased (Figures 2C–E). The defect-free nanofibers with an average diameter (560 ± 88 nm) were prepared in the electrospinning of the FD/PEO (90:10) solution (Figure 2F). Figure 2G showed the variation in viscosity with the weight ratios of FD/PEO solutions. By adding PEO with different ratios relative to FD (FD/PEO = 98/2, 95/5, and 90/10), it was found that the viscosity of solutions was increased from 0.0114 to 0.0879 Pa·s. It was reported that the chain entanglements caused by the increased polymer concentration can play a vital role in fiber formation during electrospinning (Shenoy et al., 2005; Zhou et al., 2013).

Quantification shows that the fiber diameter first decreased and then increased with increasing the amount of PEO (Figure 2H). The size of microbeads initially increased with increasing the amount of PEO and then was relatively independent of the amount of PEO. The increased chain entanglements can serve to stabilize the electrospinning jet by inhibiting jet breakup, which elongated beads (Shenoy et al., 2005). These results indicate that the morphology and diameter of electrospun FD/PEO nanofibers greatly depended on the weight ratios of FD/PEO. Also, UHMWPEO as the co-spinning polymer significantly improved the spinnability of FD.

**FIGURE 3:**
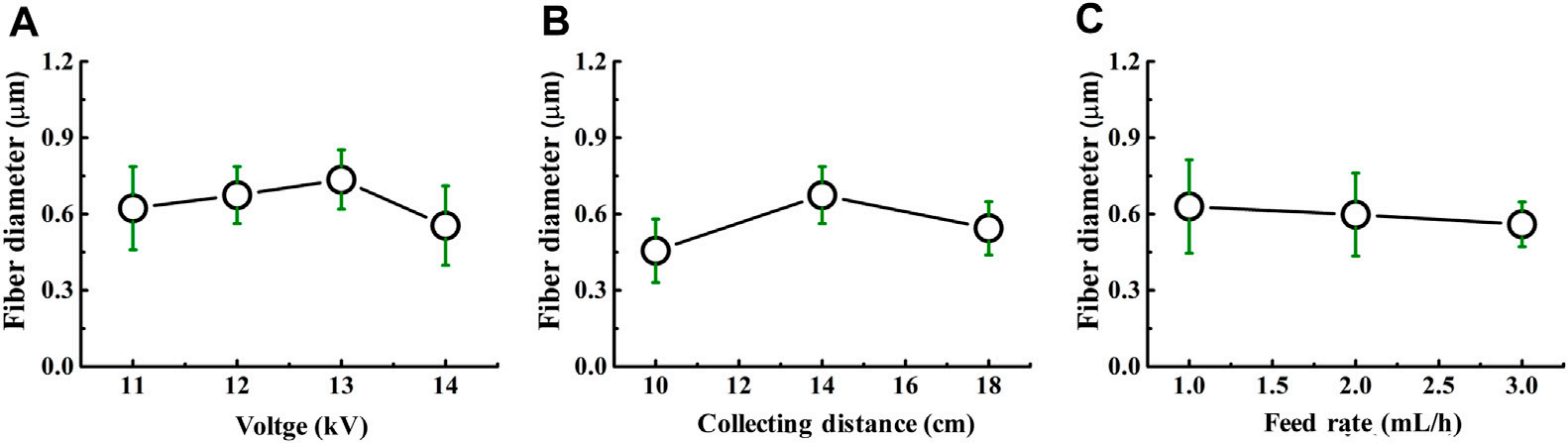
Dependence of the fiber diameter on **(A)** voltage, **(B)** collecting distance, and **(C)** feed rate.

It was well-demonstrated that the diameter of nanofibers can affect the drug release, modulate cell adhesion, migration, proliferation, differentiation, siRNA uptake, and gene silencing, as well as even tissue repair and regeneration (Jaiswal and Brown, 2012; Higgins et al., 2015; Pelipenko et al., 2015; Yau et al., 2015). As depicted in Figures 3A,B, the diameter of FD/PEO nanofibers slightly increased and then decreased with increasing the applied voltage and collecting distance. The nanofiber diameter remained unchanged with the increment of feed rate (Figure 3C).

### Characterization of the FD-Based Nanofibers

**FIGURE 4:**
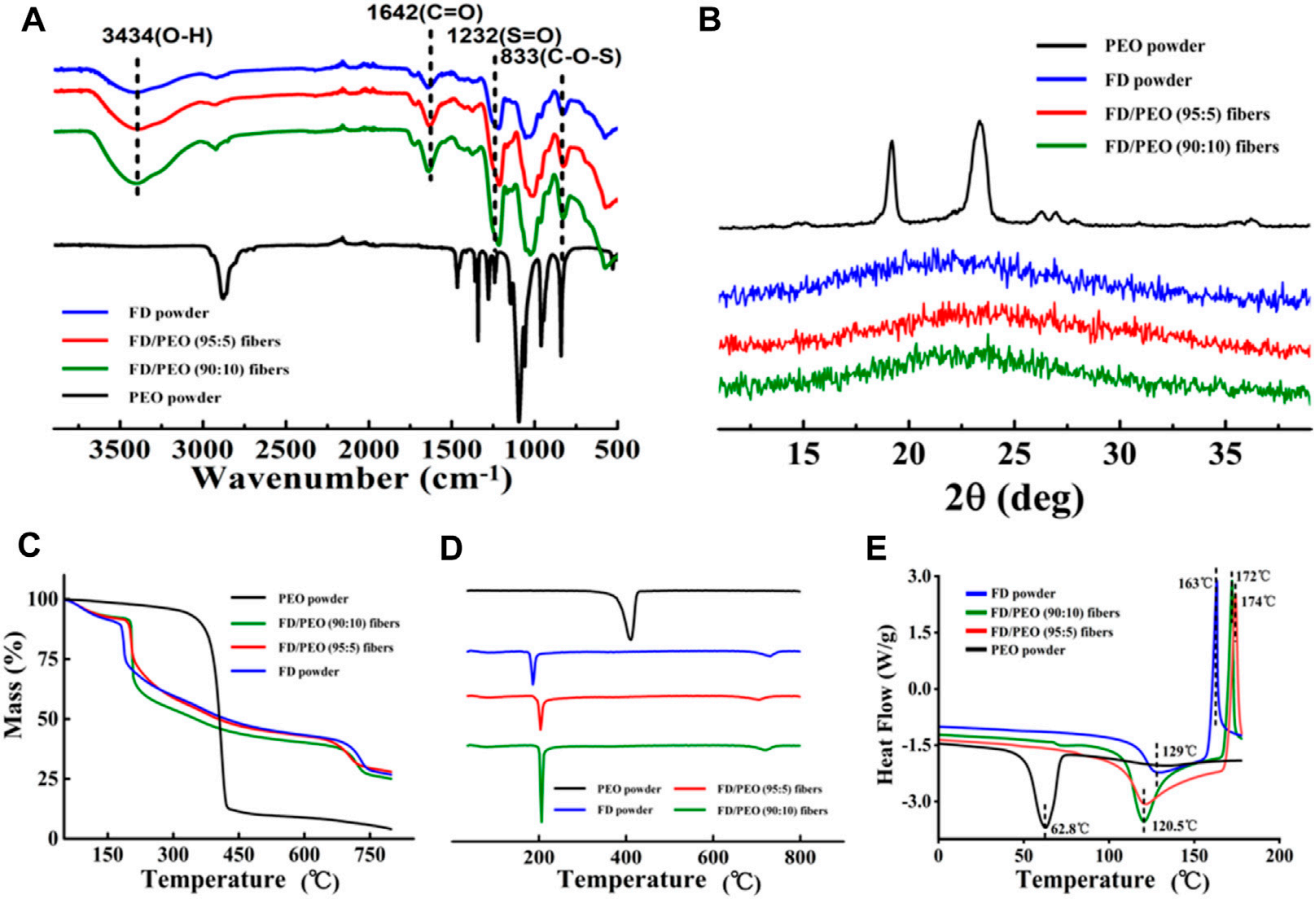
**(A)** FT-IR spectra, **(B)** XRD diffraction patterns, **(C)** Raw TGA thermograms and **(D)** their first-order derivative curves, and **(E)** DSC curves of FD powder, PEO powder, FD/PEO (95/5) beaded nanofibers, and FD/PEO (90:10) nanofibers.

FT-IR spectra were performed to ascertain the molecular interactions in FD/PEO nanofibers (Figure 4A). PEO revealed a relatively sharp peak at 2,938 cm^−1^, which is attributed to—CH_2_ stretching (Shariful et al., 2017). And its typical peaks at 1,148 and 1,110 cm^−1^ correspond to C-O-C vibration. In addition, FD showed absorption bands at 3,434 cm^−1^ (O-H stretching), 1,642 cm^−1^ (C=O stretching), 1,232 cm^−1^ (S=O bending), and 833 cm^−1^ (C-O-S bending). The absorption band associated with C-O-C disappeared in FD/PEO nanofibers, probably because C-O-C is a proton acceptor and may form hydrogen bonding with the OH group in FD molecules (Kondo et al., 1994).

Figure 4B displays the XRD patterns of raw materials and the beaded nanofibers (FD/PEO = 95:5), nanofibers (FD/PEO = 90:10). The PEO powder showed two characteristic diffraction peaks at 19.2 and 23.3°, corresponding to (120) and (112) planes, respectively. Pure FD powder at 23° displayed low overall crystallinity, which suggests that it is a semicrystalline polymer, which is consistent with other reports (Saravana et al., 2016). The XRD patterns of FD-based nanomaterials were similar to that of FD. There were no significant differences between nanomaterials with different ratios. Also, the diffraction peaks of PEO were largely depressed in the nanomaterials probably due to a small amount of added PEO and/or good miscibility between FD and PEO.

Raw TGA thermograms and their first-order derivative curves are shown in Figures 4C,D. It was found that the pure PEO is found to thermally decompose at 375°C and decomposed completely at 433°C. The FD powder showed a weight loss of approximately 28% between 35 and 200°C and a continuous weight loss until the temperature reaches 800°C. The thermal behavior of FD/PEO nanomaterials displayed a similar trend to that of FD powder. The first stage of weight loss (<100 °C) was due to moisture evaporation. The second stage exhibited a sharp decrease in weight owing to the decomposition of FD. With an increased amount of PEO, the maximum decomposition rate of FD/PEO nanomaterials slightly increased from 186 to 206°C. The reason may be due to a small amount of added PEO in composite nanofibers. Also, the introduction of PEO increased the thermal stability of FD/PEO nanomaterials.

Moreover, DSC analysis of the prepared FD/PEO nanomaterials displayed shifts in glass transition temperature with the incorporation of PEO to FD. No extra transition signals appeared as compared to the DSC curve of FD. Taken together, these results indicate that there was good miscibility or no obvious phase separation between FD and PEO.

### FD/PEO Nanofibers Modified by CS

**FIGURE 5:**
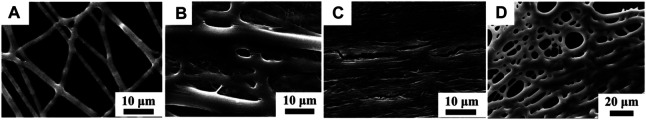
SEM images of CS-modified FD nanofibers with different weight ratios of HAc/H_2_O [i.e., **(A)** 30%, **(B)** 60%, and **(C)** 90%]. **(D)** The SEM image of CS-modified FD-based nanofibers in HAc/H_2_O = 30% after infiltrating with H_2_O.

Because PEO and FD have a high solubility in water, the structure of prepared FD/PEO fibrous membranes in the aqueous environment can be destroyed. To maintain the structure of FD/PEO nanofibers in the cell culture medium, it is necessary to modify the nanofiber surface with an H_2_O-insoluble polymer. Here, positively charged CS was selected which could interact with negatively charged FD *via* the electrostatic interaction. The FD/PEO nanofibers were soaked in 2 wt% CS solutions with various HAc/H_2_O percentages (i.e., 30, 60, and 90%). Representative SEM images of FD/PEO nanofibers before and after modification are shown in Figure 5. After the treatment of CS solution in HAc/H_2_O = 30 wt%, the integrity of the fiber structure was retained (Figure 5A). After the modification of CS solution in HAc/H_2_O = 60 and 90 wt%, the fibers swelled largely and the fiber structure was disappeared (Figures 5B,C). Next, CS-modified FD/PEO nanofibers were soaked in water for 30 min and their fiber morphology remained. However, the nanofibers had obvious swelling and adhesion (Figure 5D). Although chemical crosslinking has been widely used to make natural polymers stable, the crosslinkers used are cytotoxic. Meanwhile, the chemical crosslinking of fucoidan has been not reported. Therefore, positively charged CS was selected which could interact with negatively charged FD *via* the electrostatic interaction.

### HUVEC Attachment and Their Interactions With Monocytes

**FIGURE 6:**
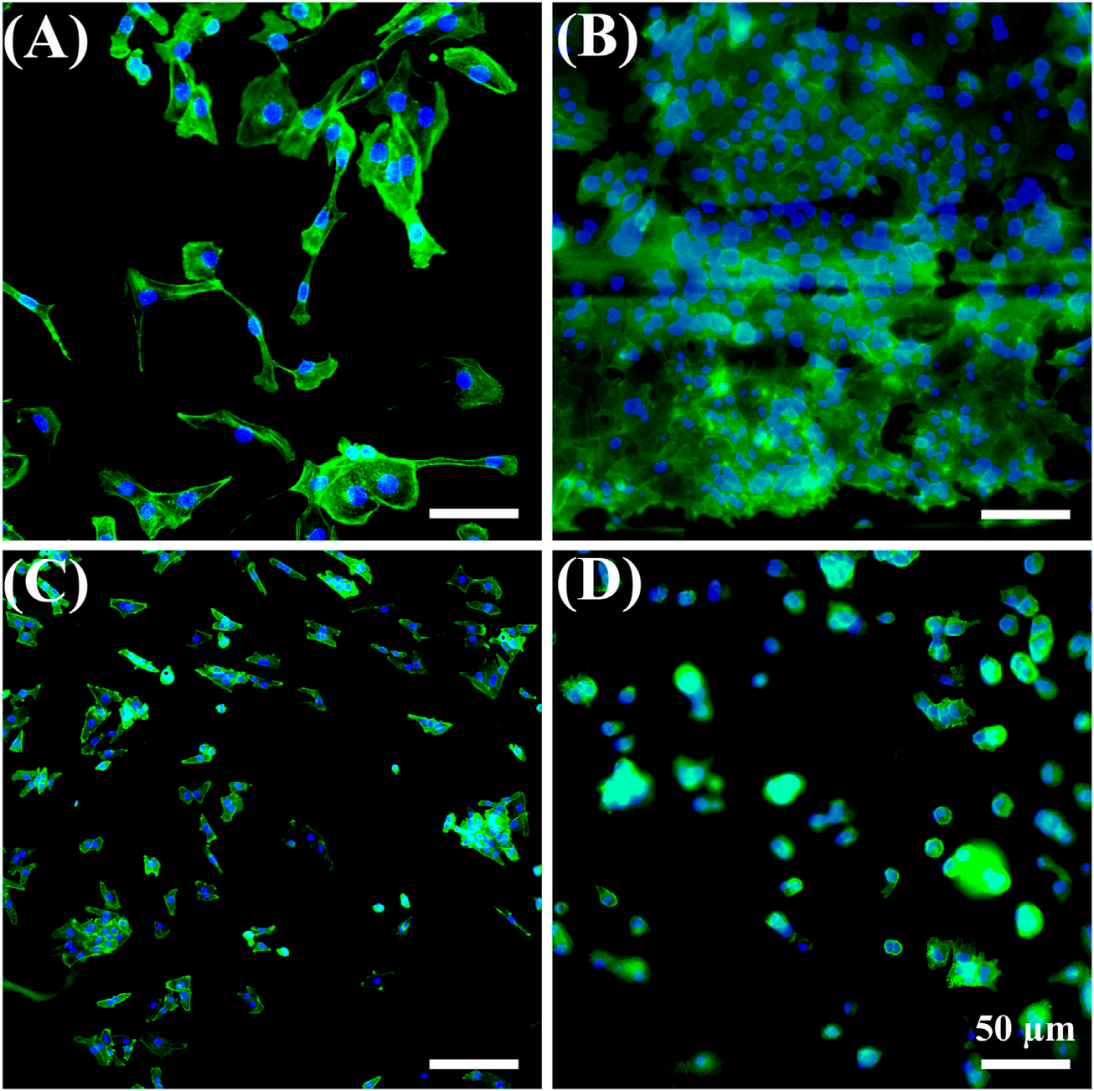
Fluorescent images of HUVECs for 1 day on the **(A)** coverslip control, **(B)** CS-modified FD/UHMWPEO nanofibers, **(C)** CS/UHMWPEO nanofibers, and **(D)** FD/CS/UHMWPEO nonfibrous film. Scale bars = 50 μm.

HUVECs were selected because they are the main cell type and play a critical role in the function of the blood vessel (Yang et al., 2017; Kang et al., 2019; Rocha et al., 2020). Cell attachment is regarded as the first and critical response of cells with their surrounding bio-scaffold, which precedes all other cellular events, e.g., survival, viability, function, and differentiation (Zhou et al., 2015; Zhou et al., 2020). As shown in Figure 6 **,** HUVEC adhesion in all samples after 1 day of cell culture was studied with a double-label fluorescence staining of the nucleus (blue) and actin cytoskeleton (green). More adhered cells were found on the CS-modified FD/UHMWPEO nanofibers compared to the CS/UHMWPEO nanofibers and FD/CS/UHMWPEO nonfibrous films, indicating that FD and fiber structure could greatly promote cell adhesion. This result suggests that the CS-modified FD/UHMWPEO nanofibers possessed excellent cytocompatibility as a bio-scaffold for blood vessel tissue engineering.

**FIGURE 7:**
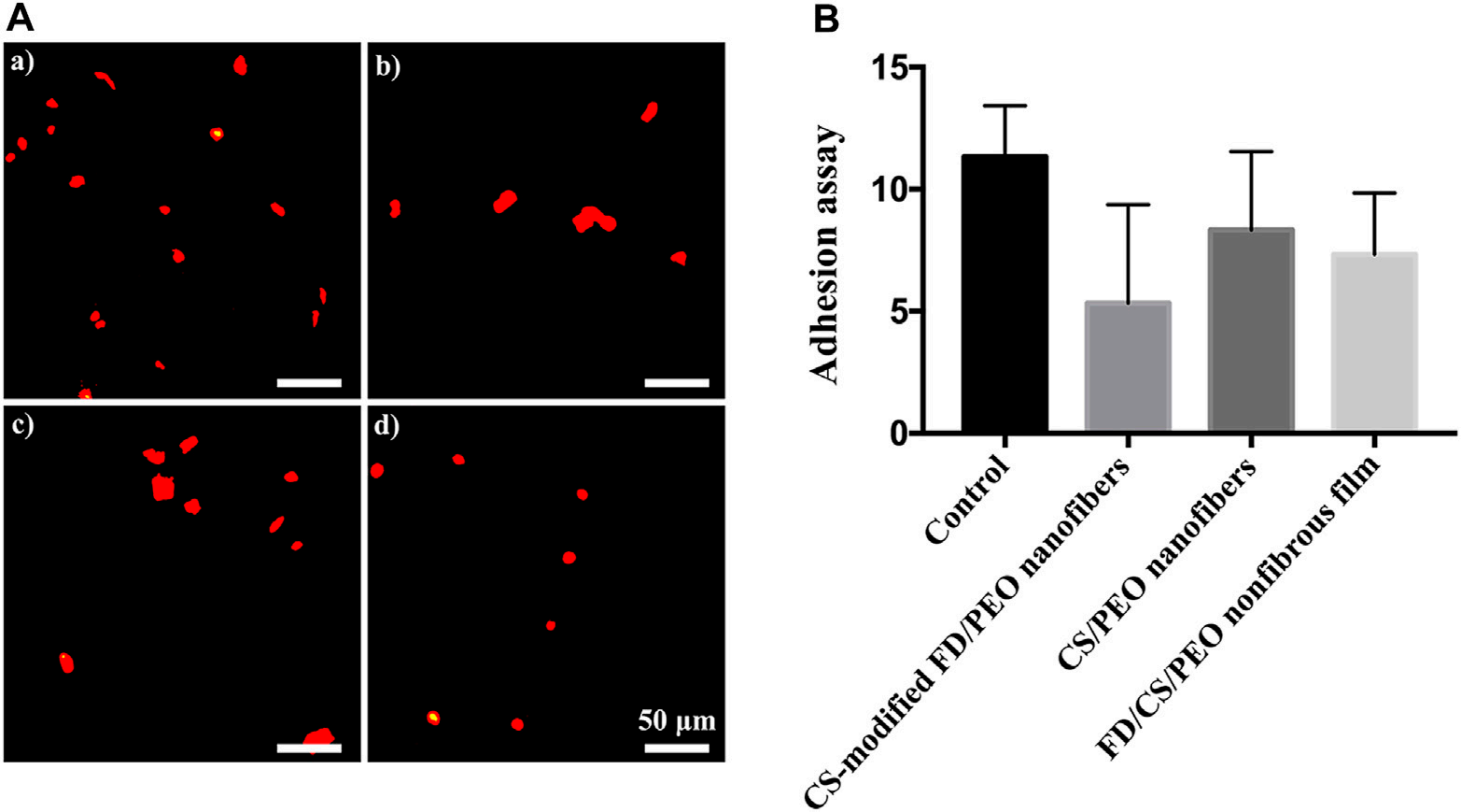
**(A)** Fluorescent images of THP-1 cells on HUVECs cultured on the (a) the coverslip control, (b) CS-modified FD/UHMWPEO nanofibers, (c) CS/UHMWPEO nanofibers, and (d) FD/CS/UHMWPEO nonfibrous film. Scale bar = 50 μm. **(B)** The number of THP-1 cells on HUVECs cultured on different samples.

The adhesion and migration of monocytes to endothelial cells is a process of the inflammatory response, which is mediated by specific molecules on endothelial cells and monocytes (Ross, 1999; Bian et al., 2017; Lin et al., 2018; Liu et al., 2020). THP-1 cells were seeded on HUVECs exposed to different materials. As shown in Figure 7A, the number of adhered monocytes on HUVECs cultured on the CS-modified FD/UHMWPEO nanofibers was less than those of other groups, indicating that the CS-modified FD/UHMWPEO nanofibers could inhibit the inflammatory response. Quantification shows that there were no significant differences among the samples.

## Conclusion

In summary, chitosan-modified FD/UHMWPEO nanofibers were fabricated using green electrospinning. Water was screened and used as a solvent to dissolve FD. The defect-free nanofibers with an average diameter (560 ± 88 nm) were prepared in the electrospinning of the FD/UHMWPEO (90:10) solution. The addition of UHMWPEO greatly improved the electrospinnability of the solution and thermo-stability of nanofibers. Cellular experiments demonstrated that the chitosan-modified FD/UHMWPEO nanofibers facilitate HUVEC adhesion and suppressed the attachment of monocytes. Thus, the developed FD-based nanofibers display great potential for vascular tissue engineering.

## Data Availability

The original contributions presented in the study are included in the article/Supplementary Material, further inquiries can be directed to the corresponding author.

## References

[B1] Ahmadi A. Ahmadi P. Sani M. A. Ehsani A. Ghanbarzadeh B. (2021). Functional Biocompatible Nanocomposite Films Consisting of Selenium and Zinc Oxide Nanoparticles Embedded in Gelatin/cellulose Nanofiber Matrices. Int. J. Biol. Macromolecules 175, 87–97. 10.1016/j.ijbiomac.2021.01.135 33485892

[B2] Bian T. Li H. Zhou Q. Ni C. Zhang Y. Yan F. (2017). Human β-Defensin 3 Reduces TNF-α-Induced Inflammation and Monocyte Adhesion in Human Umbilical Vein Endothelial Cells. Mediators Inflamm. 2017, 8529542. 10.1155/2017/8529542 28348463 PMC5350351

[B3] Bidarra S. J. Barrias C. C. Granja P. L. (2014). Injectable Alginate Hydrogels for Cell Delivery in Tissue Engineering. Acta Biomater. 10, 1646–1662. 10.1016/j.actbio.2013.12.006 24334143

[B4] Casasola R. Thomas N. L. Trybala A. Georgiadou S. (2014). Electrospun Poly Lactic Acid (PLA) Fibres: Effect of Different Solvent Systems on Fibre Morphology and Diameter. Polymer 55, 4728–4737. 10.1016/j.polymer.2014.06.032

[B5] Coultas L. Chawengsaksophak K. Rossant J. (2005). Endothelial Cells and VEGF in Vascular Development. Nature 438, 937–945. 10.1038/nature04479 16355211

[B6] Cui C. Chen X. Ma L. Zhong Q. Li Z. Mariappan A. (2020). Polythiourethane Covalent Adaptable Networks for Strong and Reworkable Adhesives and Fully Recyclable Carbon Fiber-Reinforced Composites. ACS Appl. Mater. Inter. 12, 47975–47983. 10.1021/acsami.0c14189 32986410

[B7] Daraeinejad Z. Shabani I. (2021). Enhancing Cellular Infiltration on Fluffy Polyaniline-Based Electrospun Nanofibers. Front. Bioeng. Biotechnol. 9, 641371. 10.3389/fbioe.2021.641371 34178954 PMC8219960

[B8] Devolder R. J. Bae H. Lee J. Kong H. (2011). Directed Blood Vessel Growth Using an Angiogenic Microfiber/microparticle Composite Patch. Adv. Mater. 23, 3139–3143. 10.1002/adma.201100823 21618617

[B9] Fernando I. P. S. Kim D. Nah J.-W. Jeon Y.-J. (2019). Advances in Functionalizing Fucoidans and Alginates (Bio)polymers by Structural Modifications: A Review. Chem. Eng. J. 355, 33–48. 10.1016/j.cej.2018.08.115

[B10] Fetz A. E. Wallace S. E. Bowlin G. L. (2021). Electrospun Polydioxanone Loaded with Chloroquine Modulates Template-Induced NET Release and Inflammatory Responses from Human Neutrophils. Front. Bioeng. Biotechnol. 9, 268. 10.3389/fbioe.2021.652055 PMC8111017 33987174

[B11] Hao Y. Zhao W. Zhang L. Zeng X. Sun Z. Zhang D. (2020). Bio-Multifunctional Alginate/Chitosan/Fucoidan Sponges with Enhanced Angiogenesis and Hair Follicle Regeneration for Promoting Full-Thickness Wound Healing. Mater. Des. 193, 108863. 10.1016/j.matdes.2020.108863

[B12] Higgins A. M. Banik B. L. Brown J. L. (2015). Geometry Sensing through POR1 Regulates Rac1 Activity Controlling Early Osteoblast Differentiation in Response to Nanofiber Diameter. Integr. Biol. 7, 229–236. 10.1039/c4ib00225c PMC4323730 25539497

[B13] Jaiswal D. Brown J. L. (2012). Nanofiber Diameter-Dependent MAPK Activity in Osteoblasts. J. Biomed. Mater. Res. 100A, 2921–2928. 10.1002/jbm.a.34234 22700490

[B14] Jana S. Gandhi A. Roy C. (2020). “Marine Biomaterials‐Based Systems,” in Marine Biomaterials-Based Systems. Editor Kim S-K (John Wiley & Sons Ltd), 1141–1174 . 10.1002/9781119143802.ch47

[B15] Kang P. L. Huang H. H. Chen T. Ju K. C. Kuo S. M. (2019). Angiogenesis-promoting Effect of LIPUS on hADSCs and HUVECs Cultured on Collagen/hyaluronan Scaffolds. Mater. Sci. Eng. C 102, 22–33. 10.1016/j.msec.2019.04.045 31146993

[B16] Kenry Lim C. T. (2017). Nanofiber Technology: Current Status and Emerging Developments. Prog. Polym. Sci. 70, 1–17. 10.1016/j.progpolymsci.2017.03.002

[B17] Kondo T. Sawatari C. Manley R. S. J. Gray D. G. (1994). Characterization of Hydrogen Bonding in Cellulose-Synthetic Polymer Blend Systems with Regioselectively Substituted Methylcellulose. Macromolecules 27, 210–215. 10.1021/ma00079a031

[B18] Li B. Lu F. Wei X. Zhao R. (2008). Fucoidan: Structure and Bioactivity. Molecules 13, 1671–1695. 10.3390/molecules13081671 18794778 PMC6245444

[B19] Li Q. Wang X. Lou X. Yuan H. Tu H. Li B. (2015). Genipin-crosslinked Electrospun Chitosan Nanofibers: Determination of Crosslinking Conditions and Evaluation of Cytocompatibility. Carbohydr. Polym. 130, 166–174. 10.1016/j.carbpol.2015.05.039 26076613

[B20] Li W. Yan Z. Ren J. Qu X. (2018). Manipulating Cell Fate: Dynamic Control of Cell Behaviors on Functional Platforms. Chem. Soc. Rev. 47, 8639–8684. 10.1039/c8cs00053k 30283962

[B21] Li D. Yang Y. Wang S. He X. Liu M. Bai B. (2021a). Role of Acetylation in Doxorubicin-Induced Cardiotoxicity. Redox Biol. 46, 102089. 10.1016/j.redox.2021.102089 34364220 PMC8350499

[B22] Li X. Yang Y. Wang Z. Jiang S. Meng Y. Song X. (2021b). Targeting Non-Coding RNAs in Unstable Atherosclerotic Plaques: Mechanism, Regulation, Possibilities, and Limitations. Int. J. Biol. Sci. 17, 3413–3427. 10.7150/ijbs.62506 34512156 PMC8416736

[B23] Lin Z. Jin J. Bai W. Li J. Shan X. (2018). Netrin-1 Prevents the Attachment of Monocytes to Endothelial Cells via an Anti-inflammatory Effect. Mol. Immunol. 103, 166–172. 10.1016/j.molimm.2018.08.021 30290313

[B24] Liu Y. Deng W. Yang L. Fu X. Wang Z. van Rijn P. (2020). Biointerface Topography Mediates the Interplay between Endothelial Cells and Monocytes. RSC Adv. 10, 13848–13854. 10.1039/d0ra00704h PMC9051607 35492981

[B25] Liu L. Han Z. An F. Gong X. Zhao C. Zheng W. (2021). Aptamer-Based Biosensors for the Diagnosis of Sepsis. J. Nanobiotechnology 19, 1–22. 10.1186/s12951-021-00959-5 34281552 PMC8287673

[B26] Lv D. Zhu M. Jiang Z. Jiang S. Zhang Q. Xiong R. (2018). Green Electrospun Nanofibers and Their Application in Air Filtration. Macromol. Mater. Eng. 303, 1–18. 10.1002/mame.201800336

[B27] Mooney D. J. Baldwin D. F. Suh N. P. Vacanti J. P. Langer R. (1996). Novel Approach to Fabricate Porous Sponges of Poly(d,l-Lactic-Co-Glycolic Acid) without the Use of Organic Solvents. Biomaterials 17, 1417–1422. 10.1016/0142-9612(96)87284-x 8830969

[B28] Oka S. Okabe M. Tsubura S. Mikami M. Imai A. (2020). Properties of Fucoidans Beneficial to Oral Healthcare. Odontology 108, 34–42. 10.1007/s10266-019-00437-3 31214896

[B29] Pelipenko J. Kocbek P. Kristl J. (2015). Nanofiber Diameter as a Critical Parameter Affecting Skin Cell Response. Eur. J. Pharm. Sci. 66, 29–35. 10.1016/j.ejps.2014.09.022 25301202

[B30] Peng W. Ren S. Zhang Y. Fan R. Zhou Y. Li L. (2021). MgO Nanoparticles-Incorporated PCL/Gelatin-Derived Coaxial Electrospinning Nanocellulose Membranes for Periodontal Tissue Regeneration. Front. Bioeng. Biotechnol. 9, 216. 10.3389/fbioe.2021.668428 PMC8026878 33842452

[B31] Rajendran P. Rengarajan T. Thangavel J. Nishigaki Y. Sakthisekaran D. Sethi G. (2013). The Vascular Endothelium and Human Diseases. Int. J. Biol. Sci. 9, 1057–1069. 10.7150/ijbs.7502 24250251 PMC3831119

[B32] Rocha L. A. Gomes E. D. Afonso J. L. Granja S. Baltazar F. Silva N. A. (2020). *In Vitro* evaluation of ASCs and HUVECs Co-cultures in 3D Biodegradable Hydrogels on Neurite Outgrowth and Vascular Organization. Front. Cel Dev. Biol. 8, 489. 10.3389/fcell.2020.00489 PMC7308435 32612997

[B33] Ross R. (1999). Atherosclerosis - An Inflammatory Disease. N. Engl. J. Med. 340, 115–126. 10.1056/nejm199901143400207 9887164

[B34] Saravana P. S. Cho Y.-J. Park Y.-B. Woo H.-C. Chun B.-S. (2016). Structural, Antioxidant, and Emulsifying Activities of Fucoidan from Saccharina Japonica Using Pressurized Liquid Extraction. Carbohydr. Polym. 153, 518–525. 10.1016/j.carbpol.2016.08.014 27561524

[B35] Senthilkumar K. Ramajayam G. Venkatesan J. Kim S.-K. Ahn B.-C. (2017). “Biomedical Applications of Fucoidan, Seaweed Polysaccharides,” in Seaweed Polysaccharides. Editors Venkatesan J Anil S Kim S.-K (Elsevier), 269–281. 10.1016/b978-0-12-809816-5.00014-1

[B36] Shariful M. I. Sharif S. B. Lee J. J. L. Habiba U. Ang B. C. Amalina M. A. (2017). Adsorption of Divalent Heavy Metal Ion by Mesoporous-High Surface Area Chitosan/poly (Ethylene Oxide) Nanofibrous Membrane. Carbohydr. Polym. 157, 57–64. 10.1016/j.carbpol.2016.09.063 27987964

[B37] Shenoy S. L. Bates W. D. Frisch H. L. Wnek G. E. (2005). Role of Chain Entanglements on Fiber Formation during Electrospinning of Polymer Solutions: Good Solvent, Non-specific Polymer-Polymer Interaction Limit. Polymer 46, 3372–3384. 10.1016/j.polymer.2005.03.011

[B38] Xu C. Inai R. Kotaki M. Ramakrishna S. (2004). Aligned Biodegradable Nanofibrous Structure: A Potential Scaffold for Blood Vessel Engineering. Biomaterials 25, 877–886. 10.1016/s0142-9612(03)00593-3 14609676

[B39] Xue J. Wu T. Dai Y. Xia Y. (2019). Electrospinning and Electrospun Nanofibers: Methods, Materials, and Applications. Chem. Rev. 119, 5298–5415. 10.1021/acs.chemrev.8b00593 30916938 PMC6589095

[B40] Yang J. Hao X. Li Q. Akpanyung M. Nejjari A. Neve A. L. (2017). CAGW Peptide- and PEG-Modified Gene Carrier for Selective Gene Delivery and Promotion of Angiogenesis in HUVECs *In Vivo* . ACS Appl. Mater. Inter. 9, 4485–4497. 10.1021/acsami.6b14769 28117580

[B41] Yang L. Han D. Zhan Q. Li X. Shan P. Hu Y. (2019). Blood TfR+ Exosomes Separated by a pH-Responsive Method Deliver Chemotherapeutics for Tumor Therapy. Theranostics 9, 7680–7696. 10.7150/thno.37220 31695794 PMC6831460

[B42] Yang L. Pijuan-Galito S. Rho H. S. Vasilevich A. S. Eren A. D. Ge L. (2021a). High-Throughput Methods in the Discovery and Study of Biomaterials and Materiobiology. Chem. Rev. 121, 4561–4677. 10.1021/acs.chemrev.0c00752 33705116 PMC8154331

[B43] Yang Y. Zhao X. Yu J. Chen X. Wang R. Zhang M. (2021b). Bioactive Skin-Mimicking Hydrogel Band-Aids for Diabetic Wound Healing and Infectious Skin Incision Treatment. Bioactive Mater. 6, 3962–3975. 10.1016/j.bioactmat.2021.04.007 PMC8079829 33937595

[B44] Yao Y. Zaw A. M. Anderson D. E. J. Hinds M. T. Yim E. K. F. (2020). Fucoidan Functionalization on Poly(vinyl Alcohol) Hydrogels for Improved Endothelialization and Hemocompatibility. Biomaterials 249, 120011–120024. 10.1016/j.biomaterials.2020.120011 32304872 PMC7748769

[B45] Yau W. W. Y. Long H. Gauthier N. C. Chan J. K. Y. Chew S. Y. (2015). The Effects of Nanofiber Diameter and Orientation on siRNA Uptake and Gene Silencing. Biomaterials 37, 94–106. 10.1016/j.biomaterials.2014.10.003 25453941

[B46] Yin X. Hao Y. Lu Y. Zhang D. Zhao Y. Mei L. (2021). Bio‐Multifunctional Hydrogel Patches for Repairing Full‐Thickness Abdominal Wall Defect. Adv. Funct. Mater., 2105614. 10.1002/adfm.202105614

[B47] Yu Z. Li Q. Wang J. Yu Y. Wang Y. Zhou Q. (2020). Reactive Oxygen Species-Related Nanoparticle Toxicity in the Biomedical Field. Nanoscale Res. Lett. 15, 115. 10.1186/s11671-020-03344-7 32436107 PMC7239959

[B48] Yu J. Xu K. Chen X. Zhao X. Yang Y. Chu D. (2021). Highly Stretchable, Tough, Resilient, and Antifatigue Hydrogels Based on Multiple Hydrogen Bonding Interactions Formed by Phenylalanine Derivatives. Biomacromolecules 22, 1297–1304. 10.1021/acs.biomac.0c01788 33577294

[B49] Zhang Y. Lim C. T. Ramakrishna S. Huang Z.-M. (2005). Recent Development of Polymer Nanofibers for Biomedical and Biotechnological Applications. J. Mater. Sci. Mater. Med. 16, 933–946. 10.1007/s10856-005-4428-x 16167102

[B50] Zhang Y. Venugopal J. R. El-Turki A. Ramakrishna S. Su B. Lim C. T. (2008). Electrospun Biomimetic Nanocomposite Nanofibers of Hydroxyapatite/chitosan for Bone Tissue Engineering. Biomaterials 29 (32), 4314–4322. 10.1016/j.biomaterials.2008.07.038 18715637

[B51] Zheng W. Hao Y. Wang D. Huang H. Guo F. Sun Z. (2021). Preparation of Triamcinolone Acetonide-Loaded Chitosan/fucoidan Hydrogel and its Potential Application as an Oral Mucosa Patch. Carbohydr. Polym. 272, 118493. 10.1016/j.carbpol.2021.118493 34420748

[B52] Zhou Q. Bao M. Yuan H. Zhao S. Dong W. Zhang Y. (2013). Implication of Stable Jet Length in Electrospinning for Collecting Well-Aligned Ultrafine PLLA Fibers. Polymer 54, 6867–6876. 10.1016/j.polymer.2013.10.042

[B53] Zhou Q. Xie J. Bao M. Yuan H. Ye Z. Lou X. (2015). Engineering Aligned Electrospun PLLA Microfibers with Nano-Porous Surface Nanotopography for Modulating the Responses of Vascular Smooth Muscle Cells. J. Mater. Chem. B 3, 4439–4450. 10.1039/c5tb00051c 32262788

[B54] Zhou Q. Zhang H. Zhou Y. Yu Z. Yuan H. Feng B. (2017). Alkali-Mediated Miscibility of Gelatin/Polycaprolactone for Electrospinning Homogeneous Composite Nanofibers for Tissue Scaffolding. Macromol. Biosci. 17, 1–10. 10.1002/mabi.201700268 29068545

[B55] Zhou Q. Chen J. Luan Y. Vainikka P. A. Thallmair S. Marrink S. J. (2020). Unidirectional Rotating Molecular Motors Dynamically Interact with Adsorbed Proteins to Direct the Fate of Mesenchymal Stem Cells. Sci. Adv. 6, eaay2756. 10.1126/sciadv.aay2756 32064345 PMC6989133

[B56] Zong T. Yang Y. Lin X. Jiang S. Zhao H. Liu M. (2021). 5′-tiRNA-Cys-GCA Regulates VSMC Proliferation and Phenotypic Transition by Targeting STAT4 in Aortic Dissection. Mol. Ther. Acids. 10.1016/j.omtn.2021.07.013 PMC8413832 34513311

